# Exploring α−ψ−ϕ contractive mapping: novel fixed point theorems in complete b-metric spaces

**DOI:** 10.12688/f1000research.150979.1

**Published:** 2024-06-03

**Authors:** Tamene Raji, Nasir Ali, Gudeta Hanchalu, Fikadu Tesgera Tolasa, Berhanu Seboka

**Affiliations:** 1 Mathematics, Dambi Dollo University, Dembi Dolo, Oromia, Ethiopia; 2 Mathematics, COMSATS University Islamabad, Vehari Campus, Vehari, 61100, Pakistan; 3 Dambi Dollo University, Dembi Dolo, Oromia, Ethiopia; 4 Dambi Dollo University, Dembi Dolo, Oromia, Ethiopia; 5 Mathematics, Ambo University, Ambo, Oromia, Ethiopia

**Keywords:** complete b-metric space, Altering distance function, α-admissible maping

## Abstract

**Background:**

This paper explores α-ψ-ϕ contractive mappings, extending the field of self-map and fixed-point theorems.

**Methods:**

We analyze α-ψ-ϕ contractive mappings using rigorous mathematical proofs and logical deductions.

**Results:**

A key main result is established, supported by intuitive corollaries and practical examples, highlighting the applicability of our findings.

**Conclusions:**

Our work provides a fresh perspective on contractive mappings, simplifying complex mathematical concepts and enriching the literature on fixed-point theorems.

## 1. Introduction

Banach's principle of contraction theory, as elucidated in Ref. 1, has served as a cornerstone for numerous research endeavors, facilitating expansions across various journals. The ubiquity of this theorem underscores its significance in extending the frontiers of mathematical discourse. Such expansions often entail imposing contraction restrictions and additional constraints on the ambient spaces, thereby giving rise to a diverse array of structures, including b-metric spaces. ^2^^,^^3^ The notion of b-metric spaces, an extension of conventional metric spaces, has been instrumental in enriching the landscape of mathematical investigations. ^3^^–^^6^ This versatile framework has found application in a myriad of contexts, contributing to discussions on topological structures and yielding insights into the interplay between fixed and common fixed points. ^7^^–^^9^

In this context, our research aims to address the pressing need for a deeper understanding of α-ψ-ϕ contractive mapping within the framework of b-metric spaces. ^10^ The fundamental question we seek to explore is how the principles of contraction theory can be extended to encompass this novel mapping concept. This investigation is motivated by the observation that while traditional contraction mappings have been extensively studied, the exploration of more generalized forms, such as α-ψ-ϕ contractive mappings, remains relatively uncharted territory. Our work not only seeks to fill this gap in the literature but also aims to contribute to the advancement of mathematical theory by introducing and elucidating the properties of α-ψ-ϕ contractive mappings. Through a meticulous synthesis of internet sources, scholarly publications, and pertinent literature, we establish the theoretical framework for this novel concept.

The novelty of our approach lies in the integration of α-ψ-ϕ contractive mappings into the framework of b-metric spaces, thus expanding the scope of both contraction theory and metric space theory. This novel synthesis opens up new avenues for exploration and offers fresh insights into the dynamics of fixed point theorems in non-standard spaces. In pursuit of our objectives, we present a comprehensive analysis, bolstered by corollaries, illustrative examples, and rigorous proofs, in support of our main result. By elucidating the intricacies of α-ψ-ϕ contractive mapping within the context of b-metric spaces, we aim to provide a solid foundation for further research and to inspire new avenues of inquiry in this vibrant field of mathematical exploration.

## 2. Preliminaries

This section serves as a foundational platform for our subsequent analysis, comprising key definitions, illustrative examples, essential notations, and fundamental theorems. These elements collectively provide the necessary groundwork and contextual understanding required to establish and prove our main result.

Definition 2.1. ^2^ ^,^ ^3
^
Let X be a non-empty set and s≥1 be a given real number. A function d:X×X→[0,∞) is a b-metric on X if the given below principles are apply;

(i)
d(x,y)=0 iff x=y , ∀ x,y∈X;

(ii)
d(x,y)=d(y,x) , ∀ x,y∈X;

(iii)
d(x,z)≤s[d(x,y)+d(y,z)], ∀ x,y,z∈X.

Then, b-metric space a pair of (X,d) with coefficient *s*.

Definition 2.2. ^11
^
Let *X* be any non empty set. Let f:X→X be a self mapping on *X* and α : X × *X* →[0,∞) be mapping.

We say that f is *α*-admissible if α(x,y)≥1⇒α(fx,fy)≥1 for all x,y∈X.

Example 2.1. ^7
^
Consider X={0,1,2,3}, Let f:X→X and α:X×X →[0,∞) and d(x,y)=|x−y| such that $f_{0}=0,f_{1}=2,f_{2}=1,f_{3}=3,$ and $\left\{ \begin{array}{l} \;\alpha \left(x,y\right)=1,\text{if}\left(x,y\right)\in \left\{(0,1),(0,2),(1,1),(2,2),(1,2),(2,1),(1,3),(2,3)\right\}\\ \;\alpha \left(x,y\right)=0,\text{other wise} \end{array}\right.$ . Then f is α-admissible.

Definition 2.3. ^12
^
Consider *X* as any set that is not empty. If f:X→X and α : X× X →[0,∞) are mappings, then f is triangular *α*-admissible mapping if

(i)
f is *α*-admissible mapping;
(ii)
$\left\{ \begin{array}{c} \alpha \left(x,y\right)\geq 1\\ \alpha \left(y,z\right)\geq 1 \end{array}\right.\Rightarrow \alpha \left(x,z\right)\geq 1$ , for all x,y,z∈X .

Example 2.2. ^13
^
Let X=[0,∞) , f:X→X and α:X×X →[0,∞) are mappings and $d\left(x,y\right)={\left|x-y\right|}^{2}$

We define $\mathrm{fx}=\left\{ \begin{array}{c} \frac{x^{2}}{2},x\in \left[0,1\right]\\ x+1,x>1 \end{array}\right.$ and $\alpha \left(x,y\right)=\left\{ \begin{array}{cc} 1, & x,y\in \left[0,1\right]\\ 0, & \text{other wise}\; \end{array}\right.$

Then, f is triangular *α*-admissible.

Definition 2.4. ^14
^
A function ψ :[0,∞)→[0,∞) is referred to as an altering distance function if the given below principles are apply;

(i)
ψ is continuous and non decreasing;
(ii)
ψ (t)=0 if and only if t=0 .

where, Ψ is the set of all altering distance functions.

Example 2.4. ^15
^
Let ψ : [0,∞ ) →[0,∞ ) given by;

$\psi (t)=\{\begin{array}{ll} \frac{t}{7}, & t<3\\ \frac{t^{2}+3}{t^{2}+4t+7}, & t\geq 3 \end{array},$ then ψ is an altering distance function.

Definition 2.5. ^16
^
Assuming that a b-metric space ( X,d ) with s equal to or greater than one and f:X→X are given, then sequence $\left\{x_{n}\right\}$ with $x_{n}=f^{n}x_{0}=fx_{n-1}$ for every n∈ℕ and $x_{0}\in X$ is called a picard sequence.

Proposition 2.1. ^16
^
Suppose that a b-metric space (X,d) with s equal to or greater than one and f:X→X are given. If a picard sequence $\left\{x_{n}\right\}$ of initial point $x_{0}\in X$ fulfills;

$$d\left(x_{n},x_{n+1}\right)\leq \frac{\;d\left(x_{n-1},x_{n}\right)+d\left(x_{n-1},x_{n+1}\right)+d\left(x_{n},x_{n+1}\right)+d\left(x_{n},x_{n+2}\right)+\frac{n}{s}}{d\left(x_{n-1},x_{n}\right)+d\left(x_{n-1},x_{n+1}\right)+d\left(x_{n},x_{n+1}\right)+d\left(x_{n},x_{n+2}\right)+m}d\left(x_{n-1},x_{n}\right)$$

Whenever $m,n\in {\mathbb{R}}^{+}$ and n<m. Then $\left\{x_{n}\right\}$ is a Cauchy sequence.

Theorem 2.1. ^16
^
Assuming that a complete b-metric space (X,d) with s equal to or greater than one and f : *X* → *X* are given

$$\mathrm{sd}\left(\mathrm{fx},\mathrm{fy}\right)\leq \frac{d\left(x,\mathrm{fy}\right)+d\left(x,f^{2}y\right)+d\left(y,\mathrm{fx}\right)+d\left(y,f^{2}x\right)+n}{d\left(x,\mathrm{fx}\right)+d\left(x,f^{2}x\right)+d\left(y,\mathrm{fy}\right)+d\left(y,f^{2}y\right)+m}d\left(x,y\right)$$

for each $x,y\in X,\text{if}\;m,n\in {\mathbb{R}}^{+}$ and n<m.

Then, a fixed point of f appears.

## 3. Main Results

Definition 3.1. ^8
^
Assuming that ( X,d ) is a b-metric space with s equal to or greater than one and that α:X×X→[0,∞) are given, then f:X→X is referred to as **“** α **-** ψ **-** ϕ **contractive mapping”** if some ψ, ϕ ∈Ψ for all x,y∈X with α(x,y)≥1 implies that

ψ(sd(fx,fy))≤ψ(M(x,y))−ϕ(M(x,y)) (3.1)

where, $M\left(x,y\right)=\frac{d\left(x,\mathrm{fy}\right)+d\left(x,f^{2}y\right)+d\left(y,\mathrm{fx}\right)+d\left(y,f^{2}x\right)+n}{d\left(x,\mathrm{fx}\right)+d\left(x,f^{2}x\right)+d\left(y,\mathrm{fy}\right)+d\left(y,f^{2}y\right)+m}d\left(x,y\right)$ for all x,y∈X, if $m,n\in {\mathbb{R}}^{+}$ such that n<m and ψ(t) is greater than ϕ(t) for every *t* is greater than zero.

Theorem 3.1. Assume that (X,d) is a complete b-metric space with s greater than or equal to one, and mappings f:X→X and α:X×X→[0,∞ ) are given;

Let’s the given below principles are apply;

(i)
f is α - ψ - ϕ Contractive mapping;
(ii)
f is triangular α -admissible mapping;
(iii) let $x_{0}\in X\;$ involves $\alpha (x_{0},fx_{0}$ ) ≥1 ; and
(iv)
f is continuous mapping.

Then, a fixed point of f appears

Proof: Let $x_{0}\in X$ be given as (iii), i.e. $\alpha (x_{0},fx_{0}$ ) ≥1.

From the definition (2.6) $x_{n+1}={\mathrm{fx}}_{n}$ and $x_{n}={\mathrm{fx}}_{n-1}$ for every n∈ℕ. If $x_{n_{o}}=$ $x_{n_{o+1}}$ for $n_{o}\in \mathbb{N},$ and ${\mathrm{fx}}_{n_{o}}=$ $x_{n_{o}}$ ,then $x_{n_{o}}$ is a fixed point of f.

Next, $x_{n}$ Let is not equal with $x_{n+1}$ , then $d\left(x_{n},x_{n+1}\right)$ is also not equal to zero for every *n* belongs to ℕ

Due to the triangular α -admissible mapping in (ii);

$\alpha \left(x_{0,}fx_{0}\right)=\alpha \left(x_{0,}x_{1}\right)\geq 1⟹\alpha \left(fx_{0},fx_{1}\right)\geq 1$ ; for each $x_{0,}x_{1}\in X$ .

$\alpha \left(x_{1},fx_{1}\right)=\alpha \left(x_{1},x_{2}\right)\geq 1⟹$ $\alpha \left(fx_{1},fx_{2}\right)\geq 1$ ; for each $x_{1},x_{2}\in X.$

by continuing in this process we get;

$\alpha \left(x_{n},fx_{n}\right)=\alpha \left(x_{n},x_{n+1}\right)\geq 1⟹\alpha \left(fx_{n},fx_{n+1}\right)\geq 1$ for each n∈ℕ .

From definition (3.1), obtain;

$$\begin{array}{l} \psi \left(\mathrm{sd}\left(x_{n},x_{n+1}\right)\right)=\psi \left(\mathrm{sd}\left(fx_{n-1},fx_{n}\right)\right)\\ \;\leq \psi \left(M\left(x_{n-1},x_{n}\right)\right)-\phi \left(M\left(x_{n-1},x_{n}\right)\right)\forall n\in \mathbb{N} \end{array}$$
(3.2)

where,

$$\begin{array}{c} M\left(x_{n-1},x_{n}\right)=\frac{d\left(x_{n-1},fx_{n}\right)+d\left(x_{n-1},f^{2}x_{n}\right)+d\left(x_{n},fx_{n-1}\right)+d\left(x_{n},f^{2}x_{n-1}\right)+n}{d\left(x_{n-1},fx_{n-1}\right)+d\left(x_{n-1},f^{2}x_{n-1}\right)+d\left(x_{n},fx_{n}\right)+d\left(x_{n},f^{2}x_{n}\right)+m}d\left(x_{n-1},x_{n}\right)\\ \;=\frac{d\left(x_{n-1},x_{n+1}\right)+d\left(x_{n-1},x_{n+2}\right)+d\left(x_{n},x_{n}\right)+d\left(x_{n},x_{n+1}\right)+n}{d\left(x_{n-1},x_{n}\right)+d\left(x_{n-1},x_{n+1}\right)+d\left(x_{n},x_{n+1}\right)+d\left(x_{n},x_{n+2}\right)+m}d\left(x_{n-1},x_{n}\right)\\ \;=\frac{d\left(x_{n-1},x_{n+2}\right)+d\left(x_{n-1},x_{n+1}\right)+d\left(x_{n},x_{n+1}\right)+n\;}{d\left(x_{n-1},x_{n}\right)+d\left(x_{n-1},x_{n+1}\right)+d\left(x_{n},x_{n+1}\right)+d\left(x_{n},x_{n+2}\right)+m}d\left(x_{n-1},x_{n}\right) \end{array}$$

Thus, from (3.2) we have;

$$\begin{array}{c} \psi \left(\mathrm{sd}\left(x_{n},x_{n+1}\right)\right)\leq \psi \left(\frac{d\left(x_{n-1},x_{n+2}\right)+d\left(x_{n-1},x_{n+1}\right)+d\left(x_{n},x_{n+1}\right)+n\;}{d\left(x_{n-1},x_{n}\right)+d\left(x_{n-1},x_{n+1}\right)+d\left(x_{n},x_{n+1}\right)+d\left(x_{n},x_{n+2}\right)+m}d\left(x_{n-1},x_{n}\right)\right)\\ \;-\phi \left(\frac{d\left(x_{n-1},x_{n+2}\right)+d\left(x_{n-1},x_{n+1}\right)+d\left(x_{n},x_{n+1}\right)+n\;}{d\left(x_{n-1},x_{n}\right)+d\left(x_{n-1},x_{n+1}\right)+d\left(x_{n},x_{n+1}\right)+d\left(x_{n},x_{n+2}\right)+m}d\left(x_{n-1},x_{n}\right)\right) \end{array}$$

But, $d\left(x_{n-1},x_{n+2}\right)\;$ ≤ $s\left[d\left(x_{n-1},x_{n}\right)+d\left(x_{n},x_{n+2}\right)\right]$

$$\begin{array}{l} \psi \left(\mathrm{sd}\left(x_{n},x_{n+1}\right)\right)\leq \psi \left(\frac{s\left[d\left(x_{n-1},x_{n}\right)+d\left(x_{n},x_{n+2}\right)\right]+d\left(x_{n-1},x_{n+1}\right)+d\left(x_{n},x_{n+1}\right)+n}{d\left(x_{n-1},x_{n}\right)+d\left(x_{n-1},x_{n+1}\right)+d\left(x_{n},x_{n+1}\right)+d\left(x_{n},x_{n+2}\right)+m}d\left(x_{n-1},x_{n}\right)\right)\\ \;-\phi \left(\frac{s\left[d\left(x_{n-1},x_{n}\right)+d\left(x_{n},x_{n+2}\right)\right]+d\left(x_{n-1},x_{n+1}\right)+d\left(x_{n},x_{n+1}\right)+n}{d\left(x_{n-1},x_{n}\right)+d\left(x_{n-1},x_{n+1}\right)+d\left(x_{n},x_{n+1}\right)+d\left(x_{n},x_{n+2}\right)+m}d\left(x_{n-1},x_{n}\right)\right)\\ \psi \left(\mathrm{sd}\left(x_{n},x_{n+1}\right)\right)\leq \psi \left(\frac{s\left[d\left(x_{n-1},x_{n}\right)+d\left(x_{n-1},x_{n+1}\right)+d\left(x_{n},x_{n+1}\right)+d\left(x_{n},x_{n+2}\right)\right]+n\;}{d\left(x_{n-1},x_{n}\right)+d\left(x_{n-1},x_{n+1}\right)+d\left(x_{n},x_{n+1}\right)+d\left(x_{n},x_{n+2}\right)+m}d\left(x_{n-1},x_{n}\right)\right) \end{array}$$

Since, ψ is continuous and increasing, we obtain;

$$\mathrm{sd}\left(x_{n},x_{n+1}\right)\leq \frac{s\left[d\left(x_{n-1},x_{n}\right)+d\left(x_{n-1},x_{n+1}\right)+d\left(x_{n},x_{n+1}\right)+d\left(x_{n},x_{n+2}\right)\right]+n\;}{d\left(x_{n-1},x_{n}\right)+d\left(x_{n-1},x_{n+1}\right)+d\left(x_{n},x_{n+1}\right)+d\left(x_{n},x_{n+2}\right)+m}d\left(x_{n-1},x_{n}\right)$$ (3.3)

$$d\left(x_{n},x_{n+1}\right)\leq \frac{\;d\left(x_{n-1},x_{n}\right)+d\left(x_{n-1},x_{n+1}\right)+d\left(x_{n},x_{n+1}\right)+d\left(x_{n},x_{n+2}\right)+\frac{n}{s}}{d\left(x_{n-1},x_{n}\right)+d\left(x_{n-1},x_{n+1}\right)+d\left(x_{n},x_{n+1}\right)+d\left(x_{n},x_{n+2}\right)+m}d\left(x_{n-1},x_{n}\right)$$ (3.4)

Since, the sequence $\left\{d\left(x_{n},x_{n+1}\right)\right\}$ is limited from the lower. Therefore ∃ *r* ≥ 0 implies $\lim_{n\to \infty }d\left(x_{n},x_{n+1}\right)=r.$

We want to show *r* = 0.

Suppose that *r* > 0.

Now, n→∞ of the two side of (3.4), gives:

$$\begin{array}{ll} r\leq \frac{r+2\mathrm{sr}+r+2\mathrm{sr}+\frac{n}{s}}{r+2\mathrm{sr}+r+2\mathrm{sr}+m}r & \;=\frac{2r+4\mathrm{sr}+\frac{n}{s}}{\;2r+4\mathrm{sr}+m}r\\ \;\leq \frac{2\mathrm{rs}+4s^{2}r+n}{2\mathrm{rs}+4s^{2}r+m}r \end{array}$$

Since, r > 0, s ≥ 1 and n < m , $n,m\in {\mathbb{R}}^{+}$

This implies $\frac{2\mathrm{rs}+4s^{2}r+n}{2\mathrm{rs}+4s^{2}r+m}\in \left[0,1\right)$ and $\frac{2\mathrm{rs}+4s^{2}r+n}{2\mathrm{rs}+4s^{2}r+m}\;$ < 1

$r\leq \frac{2\mathrm{rs}+4s^{2}r+n}{2\mathrm{rs}+4s^{2}r+m}r<r$ , a contradiction.

Hence, $\lim_{n\to \infty }d\left(x_{n},x_{n+1}\right)=0$ (3.5). So $\left\{x_{n}\right\}\;$ is a Cauchy sequence by ( 3.4) and Using proposition (2.1).

Let’s now demonstrate the fixed point f ;-

(∃z) (z∈ *X*) implies $\;\lim_{n\to \infty }d\left(x_{n},z\right)=0$ from a complete b-metric space

So, using definition (3.1), with $x=x_{n}\;\text{and}\;y=z$ , gives;

$$\begin{array}{c} \psi \left(\mathrm{sd}\left(x_{n+1},\mathrm{fz}\right)\right)=\psi \left(\mathrm{sd}\left(fx_{n},\mathrm{fz}\right)\right)\\ \leq \psi \left(\frac{d\left(x_{n},\mathrm{fz}\right)+d\left(x_{n},f^{2}z\right)+d\left(z,fx_{n}\right)+d\left(z,f^{2}x_{n}\right)+n}{d\left(x_{n},fx_{n}\right)+d\left(x_{n},f^{2}x_{n}\right)+d\left(z,\mathrm{fz}\right)+d\left(z,f^{2}z\right)+m}d\left(x_{n},z\right)\right)\\ -\phi \left(\frac{d\left(x_{n},\mathrm{fz}\right)+d\left(x_{n},f^{2}z\right)+d\left(z,fx_{n}\right)+\left(z,f^{2}x_{n}\right)+n}{d\left(x_{n},fx_{n}\right)+d\left(x_{n},f^{2}x_{n}\right)+d\left(z,\mathrm{fz}\right)+d\left(z,f^{2}z\right)+m}d\left(x_{n},z\right)\right) \end{array}$$

$$\psi \left(\mathrm{sd}\left(x_{n+1},\mathrm{fz}\right)\right)\leq \psi \left(\frac{d\left(x_{n},\mathrm{fz}\right)+d\left(x_{n},f^{2}z\right)+d\left(z,x_{n+1}\right)+d\left(z,x_{n+2}\right)+n}{d\left(x_{n},x_{n+1}\right)+d\left(x_{n},x_{n+2}\right)+d\left(z,\mathrm{fz}\right)+d\left(z,f^{2}z\right)+m}d\left(x_{n},z\right)\right)$$

$$\mathrm{sd}\left(x_{n+1},\mathrm{fz}\right)\leq \frac{d\left(x_{n},\mathrm{fz}\right)+d\left(x_{n},f^{2}z\right)+d\left(z,x_{n+1}\right)+d\left(z,x_{n+2}\right)+n}{d\left(x_{n},x_{n+1}\right)+d\left(x_{n},x_{n+2}\right)+d\left(z,\mathrm{fz}\right)+d\left(z,f^{2}z\right)+m}d\left(x_{n},z\right)$$
(3.6)

Taking the limit as n→∞ of the two side of (3.6), gives:

$$\lim_{n\to \infty }\left(\mathrm{sd}\left(x_{n+1},\mathrm{fz}\right)\right)\leq \lim_{n\to \infty }\left(\frac{d\left(x_{n},\mathrm{fz}\right)+d\left(x_{n},f^{2}z\right)+d\left(z,x_{n+1}\right)+d\left(z,x_{n+2}\right)+n}{d\left(x_{n},x_{n+1}\right)+d\left(x_{n},x_{n+2}\right)+d\left(z,\mathrm{fz}\right)+d\left(z,f^{2}z\right)+m}d\left(x_{n},z\right)\right)$$

$$0\leq s\lim_{n\to \infty }d\left(x_{n+1},\mathrm{fz}\right)\leq \lim_{n\to \infty }\left(\frac{d\left(x_{n},\mathrm{fz}\right)+d\left(x_{n},f^{2}z\right)+d\left(z,x_{n+1}\right)+d\left(z,x_{n+2}\right)+n}{d\left(x_{n},x_{n+1}\right)+d\left(x_{n},x_{n+2}\right)+d\left(z,\mathrm{fz}\right)+d\left(z,f^{2}z\right)+m}d\left(x_{n},z\right)\right)=0$$

$$0\leq s\lim_{n\to \infty }d\left(x_{n+1},\mathrm{fz}\right)\leq 0$$

$$s\lim_{n\to \infty }d\left(x_{n+1},\mathrm{fz}\right)=0$$

$$\lim_{n\to \infty }d\left(x_{n+1},\mathrm{fz}\right)=0$$

$$\lim_{n\to \infty }x_{n+1}=\mathrm{fz}$$

By the limit’s uniqueness and the Complete b-metric space it gives;

$$\lim_{n\to \infty }x_{n+1}=\lim_{n\to \infty }x_{n}$$

fz=z

Hence, the fixed point of f is z .

Theorem 3.2. Assume that (X,d) is a complete b-metric space with s greater than or equal to one, and that f:X→X and α:X×X→[0,∞ ) are given.

Let’s the given below principles are apply:-

(i)
f is α - ψ - ϕ Contractive mapping;
(ii)
f is triangular α -admissible mapping;
(iii)
$x_{0}\in X\;$ implies $(x_{0},fx_{0}$ ) ≥1 ;
(iv) if $\left\{x_{n}\right\}$ in X implies $(x_{n},x_{n+1}$ ) is equal to or greater than one for every n∈ℕ and $x_{n}\to x^{*}\in X$ as n→∞ , then there exist a subsequence $\left\{x_{n_{k}}\right\}$ of $\left\{x_{n}\right\}$ so that $\alpha (x_{n_{k}},x^{*}$ ) ≥1 for every k∈ℕ .

Then, a fixed point of f appears.

**Proof:** Proceed analogously to the proof of first theorem and use (iv) $(x_{n_{k}},x^{*}$ ) ≥1 for a subsequence $\left\{x_{n_{k}}\right\}$ of $\left\{x_{n}\right\}$ and k∈ℕ, we get;

$$\begin{array}{l} \psi \left(\mathrm{sd}\left(x_{n_{k+1}},fx^{*}\right)\right)=\psi \left(\mathrm{sd}\left(fx_{n_{k}},fx^{*}\right)\right)\\ \;\leq \psi \left(\frac{d\left(x_{n_{k}},fx^{*}\right)+d\left(x_{n_{k}},f^{2}x^{*}\right)+d\left(x^{*},fx_{n_{k}}\right)+d\left(x^{*},f^{2}x_{n_{k}}\right)+n}{d\left(x_{n_{k}},fx_{n_{k}}\right)+d\left(x_{n_{k}},f^{2}x_{n_{k}}\right)+d\left(x^{*},fx^{*}\right)+d\left(x^{*},f^{2}x^{*}\right)+m}d\left(x_{n_{k}},x^{*}\right)\right)\\ \;-\phi \left(\frac{d\left(x_{n_{k}},fx^{*}\right)+d\left(x_{n_{k}},f^{2}x^{*}\right)+d\left(x^{*},fx_{n_{k}}\right)+\left(x^{*},f^{2}x_{n_{k}}\right)+n}{d\left(x_{n_{k}},fx_{n_{k}}\right)+d\left(x_{n_{k}},f^{2}x_{n_{k}}\right)+d\left(x^{*},fx^{*}\right)+d\left(x^{*},f^{2}x^{*}\right)+m}d\left(x_{n_{k}},x^{*}\right)\right)\\ \psi \left(\mathrm{sd}\left(x_{n_{k+1}},fx^{*}\right)\right)=\psi \left(\mathrm{sd}\left(fx_{n_{k}},fx^{*}\right)\right)\\ \;\leq \psi \left(\frac{d\left(x_{n_{k}},fx^{*}\right)+d\left(x_{n_{k}},f^{2}x^{*}\right)+d\left(x^{*},x_{n_{k+1}}\right)+d\left(x^{*},x_{n_{k+2}}\right)+n}{d\left(x_{n_{k}},x_{n_{k+1}}\right)+d\left(x_{n_{k}},x_{n_{k+2}}\right)+d\left(x^{*},fx^{*}\right)+d\left(x^{*},f^{2}x^{*}\right)+m}d\left(x_{n_{k}},x^{*}\right)\right)\\ \;-\phi \left(\frac{d\left(x_{n_{k}},fx^{*}\right)+d\left(x_{n_{k}},f^{2}x^{*}\right)+d\left(x^{*},x_{n_{k+1}}\right)+d\left(x^{*},x_{n_{k+2}}\right)+n}{d\left(x_{n_{k}},x_{n_{k+1}}\right)+d\left(x_{n_{k}},x_{n_{k+2}}\right)+d\left(x^{*},fx^{*}\right)+d\left(x^{*},f^{2}x^{*}\right)+m}d\left(x_{n_{k}},x^{*}\right)\right)\\ \psi \left(\mathrm{sd}\left(x_{n_{k+1}},fx^{*}\right)\right)\leq \psi \left(\frac{d\left(x_{n_{k}},fx^{*}\right)+d\left(x_{n_{k}},f^{2}x^{*}\right)+d\left(x^{*},x_{n_{k+1}}\right)+d\left(x^{*},x_{n_{k+2}}\right)+n}{d\left(x_{n_{k}},x_{n_{k+1}}\right)+d\left(x_{n_{k}},x_{n_{k+2}}\right)+d\left(x^{*},fx^{*}\right)+d\left(x^{*},f^{2}x^{*}\right)+m}d\left(x_{n_{k}},x^{*}\right)\right)\\ \;\mathrm{sd}\left(x_{n_{k+1}},fx^{*}\right)\leq \frac{d\left(x_{n_{k}},fx^{*}\right)+d\left(x_{n_{k}},f^{2}x^{*}\right)+d\left(x^{*},x_{n_{k+1}}\right)+d\left(x^{*},x_{n_{k+2}}\right)+n}{d\left(x_{n_{k}},x_{n_{k+1}}\right)+d\left(x_{n_{k}},x_{n_{k+2}}\right)+d\left(x^{*},fx^{*}\right)+d\left(x^{*},f^{2}x^{*}\right)+m}d\left(x_{n_{k}},x^{*}\right) \end{array}$$
(3.7)

Taking the limit as k→∞ on two side of (3.7), gives:-

$$\lim_{k\to \infty }\left(\mathrm{sd}\left(x_{n_{k+1}},fx^{*}\right)\right)\leq \lim_{k\to \infty }\left(\frac{d\left(x_{n_{k}},fx^{*}\right)+d\left(x_{n_{k}},f^{2}x^{*}\right)+d\left(x^{*},x_{n_{k+1}}\right)+d\left(x^{*},x_{n_{k+2}}\right)+n}{d\left(x_{n_{k}},x_{n_{k+1}}\right)+d\left(x_{n_{k}},x_{n_{k+2}}\right)+d\left(x^{*},fx^{*}\right)+d\left(x^{*},f^{2}x^{*}\right)+m}d\left(x_{n_{k}},x^{*}\right)\right)$$

$$0\leq s\lim_{k\to \infty }\;\lim_{k\to \infty }\left(\frac{d\left(x_{n_{k}},fx^{*}\right)+d\left(x_{n_{k}},f^{2}x^{*}\right)+d\left(x^{*},x_{n_{k+1}}\right)+d\left(x^{*},x_{n_{k+2}}\right)+n}{d\left(x_{n_{k}},x_{n_{k+1}}\right)+d\left(x_{n_{k}},x_{n_{k+2}}\right)+d\left(x^{*},fx^{*}\right)+d\left(x^{*},f^{2}x^{*}\right)+m}d\left(x_{n_{k}},x^{*}\right)\right)=0$$

$$0\leq s\lim_{k\to \infty }d\left(x_{n_{k+1}},fx^{*}\right)\leq 0$$

$$s\lim_{k\to \infty }d\left(x_{n_{k+1}},fx^{*}\right)=0$$

$$\lim_{k\to \infty }d\left(x_{n_{k+1}},fx^{*}\right)=0$$

$$\lim_{k\to \infty }x_{n_{k+1}}=fx^{*}$$

By uniqueness of limit and Complete b-metric space we have;

$$\lim_{k\to \infty }x_{n_{k+1}}=\lim_{k\to \infty }x_{n_{k}}=x^{*}=fx^{*}$$

$$x^{*}=fx^{*}$$

Hence, the fixed point of f is $x^{*}.$

Corollary 3.1. Let f:X→X and α:X×X→[0,∞ ) are the predetermined mappings, while (X,d) is a complete metric space;

Let’s the given below principles are apply:-

(i)
f is α - ψ - ϕ Contractive mapping;
(ii)
f is triangular α -admissible mapping;
(iii) let $x_{0}\in X\;$ implies $\alpha (x_{0},fx_{0}$ ) ≥1 ; and
(iv) if $\left\{x_{n}\right\}$ in *X* implies $(x_{n},x_{n+1}$ ) ≥1 for every n∈ℕ and $x_{n}\to x^{*}\in X$ as n→∞,

For some subsequence $\left\{x_{n_{k}}\right\}$ of $\left\{x_{n}\right\}$ so that $\alpha (x_{n_{k}},x^{*}$ ) ≥1 for every k∈ℕ .

Then, a fixed point of f appears

Proof: If s is one, From Theorems **3.1** and **3.2** One finds this consequence.

Example 3.1. If *X* = {0,3,4}, d:X×X →[0,∞) is given by $d\left(x,y\right)={\left|x-y\right|}^{2},\left(X,d\right)$ is a complete b-metric space with s = 2, and also α:X×X →[0,∞) and f:X→X are given as follows; $\left\{ \begin{array}{ll} \alpha \left(x,y\right)=1, & \text{if}\;x,y\in \left\{\;0,3,4\right\}\;\\ \;\alpha \left(x,y\right)=0, & \text{other wise}\; \end{array}\right.$

f0=0,f3=3,f4=4

again, If ψ(t)=t and $\phi \left(t\right)=\frac{t}{3}$ , whenever ψ,ϕ:[0,∞)→[0,∞)∀t∈[0,∞) , then f is α - ψ - ϕ Contractive Mapping.

First, show that f is *α*-admissible mapping.

If x = 0, y=3 , α(0,3)≥1⇒α(f0,f3)=α(0,3)=1≥1

⇒f is *α*-admissible mapping.

Secondly, If x = 0, y=3andz=4 , the given equation holds.

⇒ f is triangular *α*-admissible.

Lastly, check that f is α - ψ - ϕ Contractive Mapping;

ψ(sd(fx,fy))=ψ(2d(f0,f3))=ψ(2d(0,3))=ψ(18)=18 (*)

$$\begin{array}{l} \psi \left(M\left(0,3\right)\right)-\phi \left(M\left(0,3\right)\right)=\psi \left(\frac{324+9n}{m}\right)-\phi \left(\frac{324+9n}{m}\right)\\ \;=\frac{324+9n}{m}-\frac{324+9n}{3m},\;\text{choose}\;n=\frac{1}{2}\;\text{and}\;m=1.\\ \;=\frac{324+\frac{9}{2}}{1}-\frac{324+\frac{9}{2}}{3}\\ \;=\frac{648+9}{2}-\frac{648+9}{6}=\frac{657}{2}-\frac{657}{6}\\ \;=\frac{657\;\left(3\right)-657}{6}=\frac{1,971-657}{6}\\ \;=\frac{1,314}{6}=219 \end{array}$$ (**)

From, (*) and (**), ψ(2d(f0,f3))≤ψ(M(0,3))−ϕ(M(0,3))

18 < 219

⇒ f is α - ψ - ϕ Contractive Mapping.

## Conclusion

In conclusion, this research has introduced the concept of α-ψ-ϕ contractive mapping within b-metric spaces, expanding upon contraction theory principles. Through rigorous analysis and illustrative examples, we've highlighted its significance and applicability. Future directions include exploring stability in iterative methods, investigating connections with other mathematical areas, and extending the analysis to broader classes of spaces and mappings. Overall, this exploration of α-ψ-ϕ contractive mappings opens new avenues for research with broad potential implications.

## Authors contribution

All the authors equally contributed towards this work *.*

## Data Availability

The data is provided on request to the authors.
