# Exploring α−ψ−ϕ contractive mapping: novel fixed point theorems in complete b-metric spaces

**DOI:** 10.12688/f1000research.150979.2

**Published:** 2024-12-16

**Authors:** Tamene Raji, Nasir Ali, Maysoon Qousini, Gudeta Hanchalu, Fikadu Tesgera Tolasa, Berhanu Seboka

**Affiliations:** 1 Mathematics, Dambi Dollo University, Dembi Dolo, Oromia, Ethiopia; 2 Mathematics, COMSATS University Islamabad, Vehari Campus, Vehari, 61100, Pakistan; 3 Faculty of Science and Information, Al-Zaytoonah University of Jordan, Amman, 11183, Jordan; 4 Dambi Dollo University, Dembi Dolo, Oromia, Ethiopia; 5 Dambi Dollo University, Dembi Dolo, Oromia, Ethiopia; 6 Mathematics, Ambo University, Ambo, Oromia, Ethiopia

**Keywords:** complete b-metric space, Altering distance function, α-admissible maping

## Abstract

This paper explores the concept of α−ψ−ϕ contractive mappings, contributing to the advancement of self-map extensions and fixed-point theorems within b-metric spaces. We introduce a new class of contractive mappings and demonstrate how they extend traditional contraction principles, offering a broader framework for analyzing fixed points in non-standard spaces. The main result of this study is a generalization of existing fixed-point theorems, supported by comprehensive corollaries, illustrative examples, and rigorous proofs. These findings provide deeper insights into the structure of b-metric spaces and open avenues for further applications in fields such as optimization and machine learning.

## 1. Introduction

Banach's principle of contraction theory, as elucidated in Ref. 1, has served as a cornerstone for numerous research endeavors, facilitating expansions across various journals. The ubiquity of this theorem underscores its significance in extending the frontiers of mathematical discourse. Such expansions often entail imposing contraction restrictions and additional constraints on the ambient spaces, thereby giving rise to a diverse array of structures, including b-metric spaces. ^2^^,^^3^ The notion of b-metric spaces, an extension of conventional metric spaces, has been instrumental in enriching the landscape of mathematical investigations. ^3^^–^^6^ This versatile framework has found application in a myriad of contexts, contributing to discussions on topological structures and yielding insights into the interplay between fixed and common fixed points. ^7^^–^^9^

In this context, our research aims to address the pressing need for a deeper understanding of α−ψ−ϕ contractive mapping within the framework of b-metric spaces. ^10^ The fundamental question we seek to explore is how the principles of contraction theory can be extended to encompass this novel mapping concept. This investigation is motivated by the observation that while traditional contraction mappings have been extensively studied, the exploration of more generalized forms, such as α−ψ−ϕ contractive mappings, remains relatively uncharted territory. Our work not only seeks to fill this gap in the literature but also aims to contribute to the advancement of mathematical theory by introducing and elucidating the properties of α−ψ−ϕ contractive mappings. Through a meticulous synthesis of internet sources, scholarly publications, and pertinent literature, we establish the theoretical framework for this novel concept.

The novelty of our approach lies in the integration of α−ψ−ϕ contractive mappings into the framework of b-metric spaces, thus expanding the scope of both contraction theory and metric space theory. This novel synthesis opens up new avenues for exploration and offers fresh insights into the dynamics of fixed point theorems in non-standard spaces. In pursuit of our objectives, we present a comprehensive analysis, bolstered by corollaries, illustrative examples, and rigorous proofs, in support of our main result. By elucidating the intricacies of α−ψ−ϕ contractive mapping within the context of b-metric spaces, we aim to provide a solid foundation for further research and to inspire new avenues of inquiry in this vibrant field of mathematical exploration.

This research aims to extend traditional contraction theory by introducing α−ψ−ϕ contractive mappings within the framework of b-metric spaces, providing a more generalized approach to fixed point theory. While contraction mappings have been extensively studied in conventional metric spaces, the generalization to b-metric spaces is significant because it allows for a broader range of applications, especially in real-world contexts where strict metric conditions, such as symmetry or the triangle inequality, may not hold exactly. This flexibility makes b-metric spaces highly relevant for practical applications in areas like optimization, dynamic systems, and computer science. Our work not only fills an existing gap in the literature by exploring these more generalized contractive conditions but also demonstrates how this framework can expand the applicability of contraction theory to more complex, real-world problems. Through rigorous analysis, we establish the theoretical foundation for α−ψ−ϕ contractive mappings, setting the stage for further research and practical applications. In addition to advancing the theoretical framework of α−ψ−ϕ contractive mappings within b-metric spaces, this research holds potential for practical applications in fields like data science and machine learning. ^8^^,^^9^ The generalization to b-metric spaces is particularly useful for analyzing algorithms in these domains, where ideal conditions such as exact distances or symmetric relationships often do not hold. For instance, clustering algorithms and optimization problems in machine learning frequently operate in non-Euclidean spaces, making the flexibility of b-metric spaces and generalized contraction mappings highly relevant. ^10^^,^^11^ By providing a more robust mathematical foundation for these non-standard spaces, our work can support more effective modeling of real-world systems, such as large-scale data sets or complex networks, ultimately contributing to improved algorithm performance and deeper insights in these fields.

## 2. Preliminaries

This section serves as a foundational platform for our subsequent analysis, comprising key definitions, illustrative examples, essential notations, and fundamental theorems. These elements collectively provide the necessary groundwork and contextual understanding required to establish and prove our main result.

Definition 2.1. ^2^ ^,^ ^3
^
Let X be a non-empty set and s≥1 be a given real number. A function d:X×X→[0,∞) is a b-metric on X if the given below principles are apply;

(i)
d(x,y)=0 iff x=y , ∀ x,y∈X;

(ii)
d(x,y)=d(y,x) , ∀ x,y∈X;

(iii)
d(x,z)≤s[d(x,y)+d(y,z)], ∀ x,y,z∈X.

Then, b-metric space a pair of (X,d) with coefficient *s*.

Definition 2.2. ^11
^
Let *X* be any non empty set. Let f:X→X be a self mapping on *X* and α : X × *X* →[0,∞) be mapping.

We say that f is *α*-admissible if α(x,y)≥1⇒α(fx,fy)≥1 for all x,y∈X.

Example 2.1. ^7
^
Consider X={0,1,2,3}, Let f:X→X and α:X×X →[0,∞) and d(x,y)=|x−y| such that $f_{0}=0,f_{1}=2,f_{2}=1,f_{3}=3,$ and $\left\{ \begin{array}{l} \;\alpha \left(x,y\right)=1,\text{if}\left(x,y\right)\in \left\{(0,1),(0,2),(1,1),(2,2),(1,2),(2,1),(1,3),(2,3)\right\}\\ \;\alpha \left(x,y\right)=0,\text{other wise} \end{array}\right.$ . Then f is α-admissible.

Definition 2.3. ^12
^
Consider *X* as any set that is not empty. If f:X→X and α : X× X →[0,∞) are mappings, then f is triangular *α*-admissible mapping if

(i)
f is *α*-admissible mapping;
(ii)
$\left\{ \begin{array}{c} \alpha \left(x,y\right)\geq 1\\ \alpha \left(y,z\right)\geq 1 \end{array}\right.\Rightarrow \alpha \left(x,z\right)\geq 1$ , for all x,y,z∈X .

Example 2.2. ^13
^
Let X=[0,∞) , f:X→X and α:X×X →[0,∞) are mappings and $d\left(x,y\right)={\left|x-y\right|}^{2}$

We define $\mathrm{fx}=\left\{ \begin{array}{c} \frac{x^{2}}{2},x\in \left[0,1\right]\\ x+1,x>1 \end{array}\right.$ and $\alpha \left(x,y\right)=\left\{ \begin{array}{cc} 1, & x,y\in \left[0,1\right]\\ 0, & \text{other wise}\; \end{array}\right.$

Then, f is triangular *α*-admissible.

Definition 2.4. ^14
^
A function ψ :[0,∞)→[0,∞) is referred to as an altering distance function if the given below principles are apply;

(i)
ψ is continuous and non decreasing;
(ii)
ψ (t)=0 if and only if t=0 .

where, Ψ is the set of all altering distance functions.

Example 2.4. ^15
^
Let ψ : [0,∞ ) →[0,∞ ) given by;

$\psi (t)=\{\begin{array}{ll} \frac{t}{7}, & t<3\\ \frac{t^{2}+3}{t^{2}+4t+7}, & t\geq 3 \end{array},$ then ψ is an altering distance function.

Definition 2.5. ^16
^
Assuming that a b-metric space ( X,d ) with s equal to or greater than one and f:X→X are given, then sequence $\left\{x_{n}\right\}$ with $x_{n}=f^{n}x_{0}=fx_{n-1}$ for every n∈ℕ and $x_{0}\in X$ is called a picard sequence.

Proposition 2.1. ^16
^
Suppose that a b-metric space (X,d) with s equal to or greater than one and f:X→X are given. If a picard sequence $\left\{x_{n}\right\}$ of initial point $x_{0}\in X$ fulfills;

$$d\left(x_{n},x_{n+1}\right)\leq \frac{\;d\left(x_{n-1},x_{n}\right)+d\left(x_{n-1},x_{n+1}\right)+d\left(x_{n},x_{n+1}\right)+d\left(x_{n},x_{n+2}\right)+\frac{n}{s}}{d\left(x_{n-1},x_{n}\right)+d\left(x_{n-1},x_{n+1}\right)+d\left(x_{n},x_{n+1}\right)+d\left(x_{n},x_{n+2}\right)+m}d\left(x_{n-1},x_{n}\right)$$

Whenever $m,n\in {\mathbb{R}}^{+}$ and n<m. Then $\left\{x_{n}\right\}$ is a Cauchy sequence.

Theorem 2.1. ^16
^
Assuming that a complete b-metric space (X,d) with s equal to or greater than one and f : *X* → *X* are given

$$\mathrm{sd}\left(\mathrm{fx},\mathrm{fy}\right)\leq \frac{d\left(x,\mathrm{fy}\right)+d\left(x,f^{2}y\right)+d\left(y,\mathrm{fx}\right)+d\left(y,f^{2}x\right)+n}{d\left(x,\mathrm{fx}\right)+d\left(x,f^{2}x\right)+d\left(y,\mathrm{fy}\right)+d\left(y,f^{2}y\right)+m}d\left(x,y\right)$$

for each $x,y\in X,\text{if}\;m,n\in {\mathbb{R}}^{+}$ and n<m.

Then, a fixed point of f appears.

## 3. Main Results

Definition 3.1. A mapping f:X→X is called a contractive mapping if there exists a constant 0<k<1 such that for all x,y∈X the following inequality holds:

d(f(x),f(y))≤k⋅d(x,y)

where (X,d) is a metric space. This condition ensures that the distance between the images of x and y under f is strictly less than the distance between x and y , thus driving points closer together.

Let (X,d) be a b-metric space with s≥1 , and let α:X×X→[0,∞) be a function defined on pairs of points in X . A mapping f:X→X is referred to as an α−ψ−ϕ contractive mapping if there exist functions ψ,ϕ∈Ψ such that for all x,y∈X with α(x,y)≥1 , the following condition is satisfied:

ψ(sd(f(x),f(y)))≤ψ(M(x,y))−ϕ(M(x,y)) (3.1)

where, $M\left(x,y\right)=d\left(x,f(y)\right)+d\left(x,f^{2}(y)\right)+d\left(y,f(x)\right)+d\left(y,f^{2}(x))+nd(x,f(x))+d(x,f^{2}(x))+d(y,f(y)\right)+d\left(y,f^{2}(y)\right)+m\cdot d(x,y)$ for all x,y∈X , with $m,n\in {\mathbb{R}}^{+}$ such that n<m and ψ(t)>ϕ(t) for every t>0 .

This definition extends the classical notion of contractive mappings by incorporating the functions ψ and ϕ that allow for more flexible contraction conditions, particularly in the context of b-metric spaces.

Theorem 3.1. Let (X,d) be a complete b-metric space with s≥1 . Consider a mapping f:X→X and a function α:X×X→[0,∞) . Assume the following conditions hold:

(i)
f is an α−ψ−ϕ contractive mapping.
(ii)
f is a triangular α -admissible mapping
(iii) There exists an initial point $x_{0}\in X,$ such that $\alpha (x_{0},fx_{0})\geq 1$ .
(iv)
f is continuous.

Then, there exists a point $x^{*}\in X$ such that $f\left(x^{*}\right)=x^{*}$ , indicating that $x^{*}$ is a fixed point of the mapping f .

Proof: Let $x_{0}\in X$ be given as (iii), i.e. $\alpha (x_{0},fx_{0}$ ) ≥1.

From the definition (2.6) $x_{n+1}={\mathrm{fx}}_{n}$ and $x_{n}={\mathrm{fx}}_{n-1}$ for every n∈ℕ. If $x_{n_{o}}=$ $x_{n_{o+1}}$ for $n_{o}\in \mathbb{N},$ and ${\mathrm{fx}}_{n_{o}}=$ $x_{n_{o}}$ ,then $x_{n_{o}}$ is a fixed point of f.

Next, $x_{n}$ Let is not equal with $x_{n+1}$ , then $d\left(x_{n},x_{n+1}\right)$ is also not equal to zero for every *n* belongs to ℕ

Due to the triangular α -admissible mapping in (ii);

$\alpha \left(x_{0,}fx_{0}\right)=\alpha \left(x_{0,}x_{1}\right)\geq 1⟹\alpha \left(fx_{0},fx_{1}\right)\geq 1$ ; for each $x_{0,}x_{1}\in X$ .

$\alpha \left(x_{1},fx_{1}\right)=\alpha \left(x_{1},x_{2}\right)\geq 1⟹$ $\alpha \left(fx_{1},fx_{2}\right)\geq 1$ ; for each $x_{1},x_{2}\in X.$

by continuing in this process we get;

$\alpha \left(x_{n},fx_{n}\right)=\alpha \left(x_{n},x_{n+1}\right)\geq 1⟹\alpha \left(fx_{n},fx_{n+1}\right)\geq 1$ for each n∈ℕ .

From definition (3.1), obtain;

$$\begin{array}{l} \psi \left(\mathrm{sd}\left(x_{n},x_{n+1}\right)\right)=\psi \left(\mathrm{sd}\left(fx_{n-1},fx_{n}\right)\right)\\ \;\leq \psi \left(M\left(x_{n-1},x_{n}\right)\right)-\phi \left(M\left(x_{n-1},x_{n}\right)\right)\forall n\in \mathbb{N} \end{array}$$
(3.2)

where,

$$\begin{array}{c} M\left(x_{n-1},x_{n}\right)=\frac{d\left(x_{n-1},fx_{n}\right)+d\left(x_{n-1},f^{2}x_{n}\right)+d\left(x_{n},fx_{n-1}\right)+d\left(x_{n},f^{2}x_{n-1}\right)+n}{d\left(x_{n-1},fx_{n-1}\right)+d\left(x_{n-1},f^{2}x_{n-1}\right)+d\left(x_{n},fx_{n}\right)+d\left(x_{n},f^{2}x_{n}\right)+m}d\left(x_{n-1},x_{n}\right)\\ \;=\frac{d\left(x_{n-1},x_{n+1}\right)+d\left(x_{n-1},x_{n+2}\right)+d\left(x_{n},x_{n}\right)+d\left(x_{n},x_{n+1}\right)+n}{d\left(x_{n-1},x_{n}\right)+d\left(x_{n-1},x_{n+1}\right)+d\left(x_{n},x_{n+1}\right)+d\left(x_{n},x_{n+2}\right)+m}d\left(x_{n-1},x_{n}\right)\\ \;=\frac{d\left(x_{n-1},x_{n+2}\right)+d\left(x_{n-1},x_{n+1}\right)+d\left(x_{n},x_{n+1}\right)+n\;}{d\left(x_{n-1},x_{n}\right)+d\left(x_{n-1},x_{n+1}\right)+d\left(x_{n},x_{n+1}\right)+d\left(x_{n},x_{n+2}\right)+m}d\left(x_{n-1},x_{n}\right) \end{array}$$

Thus, from (3.2) we have;

$$\begin{array}{c} \psi \left(\mathrm{sd}\left(x_{n},x_{n+1}\right)\right)\leq \psi \left(\frac{d\left(x_{n-1},x_{n+2}\right)+d\left(x_{n-1},x_{n+1}\right)+d\left(x_{n},x_{n+1}\right)+n\;}{d\left(x_{n-1},x_{n}\right)+d\left(x_{n-1},x_{n+1}\right)+d\left(x_{n},x_{n+1}\right)+d\left(x_{n},x_{n+2}\right)+m}d\left(x_{n-1},x_{n}\right)\right)\\ \;-\phi \left(\frac{d\left(x_{n-1},x_{n+2}\right)+d\left(x_{n-1},x_{n+1}\right)+d\left(x_{n},x_{n+1}\right)+n\;}{d\left(x_{n-1},x_{n}\right)+d\left(x_{n-1},x_{n+1}\right)+d\left(x_{n},x_{n+1}\right)+d\left(x_{n},x_{n+2}\right)+m}d\left(x_{n-1},x_{n}\right)\right) \end{array}$$

But, $d\left(x_{n-1},x_{n+2}\right)\;$ ≤ $s\left[d\left(x_{n-1},x_{n}\right)+d\left(x_{n},x_{n+2}\right)\right]$

$$\begin{array}{l} \psi \left(\mathrm{sd}\left(x_{n},x_{n+1}\right)\right)\leq \psi \left(\frac{s\left[d\left(x_{n-1},x_{n}\right)+d\left(x_{n},x_{n+2}\right)\right]+d\left(x_{n-1},x_{n+1}\right)+d\left(x_{n},x_{n+1}\right)+n}{d\left(x_{n-1},x_{n}\right)+d\left(x_{n-1},x_{n+1}\right)+d\left(x_{n},x_{n+1}\right)+d\left(x_{n},x_{n+2}\right)+m}d\left(x_{n-1},x_{n}\right)\right)\\ \;-\phi \left(\frac{s\left[d\left(x_{n-1},x_{n}\right)+d\left(x_{n},x_{n+2}\right)\right]+d\left(x_{n-1},x_{n+1}\right)+d\left(x_{n},x_{n+1}\right)+n}{d\left(x_{n-1},x_{n}\right)+d\left(x_{n-1},x_{n+1}\right)+d\left(x_{n},x_{n+1}\right)+d\left(x_{n},x_{n+2}\right)+m}d\left(x_{n-1},x_{n}\right)\right)\\ \psi \left(\mathrm{sd}\left(x_{n},x_{n+1}\right)\right)\leq \psi \left(\frac{s\left[d\left(x_{n-1},x_{n}\right)+d\left(x_{n-1},x_{n+1}\right)+d\left(x_{n},x_{n+1}\right)+d\left(x_{n},x_{n+2}\right)\right]+n\;}{d\left(x_{n-1},x_{n}\right)+d\left(x_{n-1},x_{n+1}\right)+d\left(x_{n},x_{n+1}\right)+d\left(x_{n},x_{n+2}\right)+m}d\left(x_{n-1},x_{n}\right)\right) \end{array}$$

Since, ψ is continuous and increasing, we obtain;

$$\mathrm{sd}\left(x_{n},x_{n+1}\right)\leq \frac{s\left[d\left(x_{n-1},x_{n}\right)+d\left(x_{n-1},x_{n+1}\right)+d\left(x_{n},x_{n+1}\right)+d\left(x_{n},x_{n+2}\right)\right]+n\;}{d\left(x_{n-1},x_{n}\right)+d\left(x_{n-1},x_{n+1}\right)+d\left(x_{n},x_{n+1}\right)+d\left(x_{n},x_{n+2}\right)+m}d\left(x_{n-1},x_{n}\right)$$ (3.3)

$$d\left(x_{n},x_{n+1}\right)\leq \frac{\;d\left(x_{n-1},x_{n}\right)+d\left(x_{n-1},x_{n+1}\right)+d\left(x_{n},x_{n+1}\right)+d\left(x_{n},x_{n+2}\right)+\frac{n}{s}}{d\left(x_{n-1},x_{n}\right)+d\left(x_{n-1},x_{n+1}\right)+d\left(x_{n},x_{n+1}\right)+d\left(x_{n},x_{n+2}\right)+m}d\left(x_{n-1},x_{n}\right)$$ (3.4)

Since, the sequence $\left\{d\left(x_{n},x_{n+1}\right)\right\}$ is limited from the lower. Therefore ∃ *r* ≥ 0 implies $\lim_{n\to \infty }d\left(x_{n},x_{n+1}\right)=r.$

We want to show *r* = 0.

Suppose that *r* > 0.

Now, n→∞ of the two side of (3.4), gives:

$$\begin{array}{ll} r\leq \frac{r+2\mathrm{sr}+r+2\mathrm{sr}+\frac{n}{s}}{r+2\mathrm{sr}+r+2\mathrm{sr}+m}r & \;=\frac{2r+4\mathrm{sr}+\frac{n}{s}}{\;2r+4\mathrm{sr}+m}r\\ \;\leq \frac{2\mathrm{rs}+4s^{2}r+n}{2\mathrm{rs}+4s^{2}r+m}r \end{array}$$

Since, r > 0, s ≥ 1 and n < m , $n,m\in {\mathbb{R}}^{+}$

This implies $\frac{2\mathrm{rs}+4s^{2}r+n}{2\mathrm{rs}+4s^{2}r+m}\in \left[0,1\right)$ and $\frac{2\mathrm{rs}+4s^{2}r+n}{2\mathrm{rs}+4s^{2}r+m}\;$ < 1

$r\leq \frac{2\mathrm{rs}+4s^{2}r+n}{2\mathrm{rs}+4s^{2}r+m}r<r$ , a contradiction.

Hence, $\lim_{n\to \infty }d\left(x_{n},x_{n+1}\right)=0$ (3.5). So $\left\{x_{n}\right\}\;$ is a Cauchy sequence by ( 3.4) and Using proposition (2.1).

Let’s now demonstrate the fixed point f ;-

(∃z) (z∈ *X*) implies $\;\lim_{n\to \infty }d\left(x_{n},z\right)=0$ from a complete b-metric space

So, using definition (3.1), with $x=x_{n}\;\text{and}\;y=z$ , gives;

$$\begin{array}{c} \psi \left(\mathrm{sd}\left(x_{n+1},\mathrm{fz}\right)\right)=\psi \left(\mathrm{sd}\left(fx_{n},\mathrm{fz}\right)\right)\\ \leq \psi \left(\frac{d\left(x_{n},\mathrm{fz}\right)+d\left(x_{n},f^{2}z\right)+d\left(z,fx_{n}\right)+d\left(z,f^{2}x_{n}\right)+n}{d\left(x_{n},fx_{n}\right)+d\left(x_{n},f^{2}x_{n}\right)+d\left(z,\mathrm{fz}\right)+d\left(z,f^{2}z\right)+m}d\left(x_{n},z\right)\right)\\ -\phi \left(\frac{d\left(x_{n},\mathrm{fz}\right)+d\left(x_{n},f^{2}z\right)+d\left(z,fx_{n}\right)+\left(z,f^{2}x_{n}\right)+n}{d\left(x_{n},fx_{n}\right)+d\left(x_{n},f^{2}x_{n}\right)+d\left(z,\mathrm{fz}\right)+d\left(z,f^{2}z\right)+m}d\left(x_{n},z\right)\right) \end{array}$$

$$\psi \left(\mathrm{sd}\left(x_{n+1},\mathrm{fz}\right)\right)\leq \psi \left(\frac{d\left(x_{n},\mathrm{fz}\right)+d\left(x_{n},f^{2}z\right)+d\left(z,x_{n+1}\right)+d\left(z,x_{n+2}\right)+n}{d\left(x_{n},x_{n+1}\right)+d\left(x_{n},x_{n+2}\right)+d\left(z,\mathrm{fz}\right)+d\left(z,f^{2}z\right)+m}d\left(x_{n},z\right)\right)$$

$$\mathrm{sd}\left(x_{n+1},\mathrm{fz}\right)\leq \frac{d\left(x_{n},\mathrm{fz}\right)+d\left(x_{n},f^{2}z\right)+d\left(z,x_{n+1}\right)+d\left(z,x_{n+2}\right)+n}{d\left(x_{n},x_{n+1}\right)+d\left(x_{n},x_{n+2}\right)+d\left(z,\mathrm{fz}\right)+d\left(z,f^{2}z\right)+m}d\left(x_{n},z\right)$$
(3.6)

Taking the limit as n→∞ of the two side of (3.6), gives:

$$\lim_{n\to \infty }\left(\mathrm{sd}\left(x_{n+1},\mathrm{fz}\right)\right)\leq \lim_{n\to \infty }\left(\frac{d\left(x_{n},\mathrm{fz}\right)+d\left(x_{n},f^{2}z\right)+d\left(z,x_{n+1}\right)+d\left(z,x_{n+2}\right)+n}{d\left(x_{n},x_{n+1}\right)+d\left(x_{n},x_{n+2}\right)+d\left(z,\mathrm{fz}\right)+d\left(z,f^{2}z\right)+m}d\left(x_{n},z\right)\right)$$

$$0\leq s\lim_{n\to \infty }d\left(x_{n+1},\mathrm{fz}\right)\leq \lim_{n\to \infty }\left(\frac{d\left(x_{n},\mathrm{fz}\right)+d\left(x_{n},f^{2}z\right)+d\left(z,x_{n+1}\right)+d\left(z,x_{n+2}\right)+n}{d\left(x_{n},x_{n+1}\right)+d\left(x_{n},x_{n+2}\right)+d\left(z,\mathrm{fz}\right)+d\left(z,f^{2}z\right)+m}d\left(x_{n},z\right)\right)=0$$

$$0\leq s\lim_{n\to \infty }d\left(x_{n+1},\mathrm{fz}\right)\leq 0$$

$$s\lim_{n\to \infty }d\left(x_{n+1},\mathrm{fz}\right)=0$$

$$\lim_{n\to \infty }d\left(x_{n+1},\mathrm{fz}\right)=0$$

$$\lim_{n\to \infty }x_{n+1}=\mathrm{fz}$$

By the limit’s uniqueness and the Complete b-metric space it gives;

$$\lim_{n\to \infty }x_{n+1}=\lim_{n\to \infty }x_{n}$$

fz=z

Hence, the fixed point of f is z .

Theorem 3.2. Assume that (X,d) is a complete b-metric space with s greater than or equal to one, and that f:X→X and α:X×X→[0,∞ ) are given.

Let’s the given below principles are apply:-

(i)
f is α - ψ - ϕ Contractive mapping;
(ii)
f is triangular α -admissible mapping;
(iii)
$x_{0}\in X\;$ implies $(x_{0},fx_{0}$ ) ≥1 ;
(iv) if $\left\{x_{n}\right\}$ in X implies $(x_{n},x_{n+1}$ ) is equal to or greater than one for every n∈ℕ and $x_{n}\to x^{*}\in X$ as n→∞ , then there exist a subsequence $\left\{x_{n_{k}}\right\}$ of $\left\{x_{n}\right\}$ so that $\alpha (x_{n_{k}},x^{*}$ ) ≥1 for every k∈ℕ .

Then, a fixed point of f appears.

**Proof:** Proceed analogously to the proof of first theorem and use (iv) $(x_{n_{k}},x^{*}$ ) ≥1 for a subsequence $\left\{x_{n_{k}}\right\}$ of $\left\{x_{n}\right\}$ and k∈ℕ, we get;

$$\begin{array}{l} \psi \left(\mathrm{sd}\left(x_{n_{k+1}},fx^{*}\right)\right)=\psi \left(\mathrm{sd}\left(fx_{n_{k}},fx^{*}\right)\right)\\ \;\leq \psi \left(\frac{d\left(x_{n_{k}},fx^{*}\right)+d\left(x_{n_{k}},f^{2}x^{*}\right)+d\left(x^{*},fx_{n_{k}}\right)+d\left(x^{*},f^{2}x_{n_{k}}\right)+n}{d\left(x_{n_{k}},fx_{n_{k}}\right)+d\left(x_{n_{k}},f^{2}x_{n_{k}}\right)+d\left(x^{*},fx^{*}\right)+d\left(x^{*},f^{2}x^{*}\right)+m}d\left(x_{n_{k}},x^{*}\right)\right)\\ \;-\phi \left(\frac{d\left(x_{n_{k}},fx^{*}\right)+d\left(x_{n_{k}},f^{2}x^{*}\right)+d\left(x^{*},fx_{n_{k}}\right)+\left(x^{*},f^{2}x_{n_{k}}\right)+n}{d\left(x_{n_{k}},fx_{n_{k}}\right)+d\left(x_{n_{k}},f^{2}x_{n_{k}}\right)+d\left(x^{*},fx^{*}\right)+d\left(x^{*},f^{2}x^{*}\right)+m}d\left(x_{n_{k}},x^{*}\right)\right)\\ \psi \left(\mathrm{sd}\left(x_{n_{k+1}},fx^{*}\right)\right)=\psi \left(\mathrm{sd}\left(fx_{n_{k}},fx^{*}\right)\right)\\ \;\leq \psi \left(\frac{d\left(x_{n_{k}},fx^{*}\right)+d\left(x_{n_{k}},f^{2}x^{*}\right)+d\left(x^{*},x_{n_{k+1}}\right)+d\left(x^{*},x_{n_{k+2}}\right)+n}{d\left(x_{n_{k}},x_{n_{k+1}}\right)+d\left(x_{n_{k}},x_{n_{k+2}}\right)+d\left(x^{*},fx^{*}\right)+d\left(x^{*},f^{2}x^{*}\right)+m}d\left(x_{n_{k}},x^{*}\right)\right)\\ \;-\phi \left(\frac{d\left(x_{n_{k}},fx^{*}\right)+d\left(x_{n_{k}},f^{2}x^{*}\right)+d\left(x^{*},x_{n_{k+1}}\right)+d\left(x^{*},x_{n_{k+2}}\right)+n}{d\left(x_{n_{k}},x_{n_{k+1}}\right)+d\left(x_{n_{k}},x_{n_{k+2}}\right)+d\left(x^{*},fx^{*}\right)+d\left(x^{*},f^{2}x^{*}\right)+m}d\left(x_{n_{k}},x^{*}\right)\right)\\ \psi \left(\mathrm{sd}\left(x_{n_{k+1}},fx^{*}\right)\right)\leq \psi \left(\frac{d\left(x_{n_{k}},fx^{*}\right)+d\left(x_{n_{k}},f^{2}x^{*}\right)+d\left(x^{*},x_{n_{k+1}}\right)+d\left(x^{*},x_{n_{k+2}}\right)+n}{d\left(x_{n_{k}},x_{n_{k+1}}\right)+d\left(x_{n_{k}},x_{n_{k+2}}\right)+d\left(x^{*},fx^{*}\right)+d\left(x^{*},f^{2}x^{*}\right)+m}d\left(x_{n_{k}},x^{*}\right)\right)\\ \;\mathrm{sd}\left(x_{n_{k+1}},fx^{*}\right)\leq \frac{d\left(x_{n_{k}},fx^{*}\right)+d\left(x_{n_{k}},f^{2}x^{*}\right)+d\left(x^{*},x_{n_{k+1}}\right)+d\left(x^{*},x_{n_{k+2}}\right)+n}{d\left(x_{n_{k}},x_{n_{k+1}}\right)+d\left(x_{n_{k}},x_{n_{k+2}}\right)+d\left(x^{*},fx^{*}\right)+d\left(x^{*},f^{2}x^{*}\right)+m}d\left(x_{n_{k}},x^{*}\right) \end{array}$$
(3.7)

Taking the limit as k→∞ on two side of (3.7), gives:-

$$\lim_{k\to \infty }\left(\mathrm{sd}\left(x_{n_{k+1}},fx^{*}\right)\right)\leq \lim_{k\to \infty }\left(\frac{d\left(x_{n_{k}},fx^{*}\right)+d\left(x_{n_{k}},f^{2}x^{*}\right)+d\left(x^{*},x_{n_{k+1}}\right)+d\left(x^{*},x_{n_{k+2}}\right)+n}{d\left(x_{n_{k}},x_{n_{k+1}}\right)+d\left(x_{n_{k}},x_{n_{k+2}}\right)+d\left(x^{*},fx^{*}\right)+d\left(x^{*},f^{2}x^{*}\right)+m}d\left(x_{n_{k}},x^{*}\right)\right)$$

$$0\leq s\lim_{k\to \infty }\;\lim_{k\to \infty }\left(\frac{d\left(x_{n_{k}},fx^{*}\right)+d\left(x_{n_{k}},f^{2}x^{*}\right)+d\left(x^{*},x_{n_{k+1}}\right)+d\left(x^{*},x_{n_{k+2}}\right)+n}{d\left(x_{n_{k}},x_{n_{k+1}}\right)+d\left(x_{n_{k}},x_{n_{k+2}}\right)+d\left(x^{*},fx^{*}\right)+d\left(x^{*},f^{2}x^{*}\right)+m}d\left(x_{n_{k}},x^{*}\right)\right)=0$$

$$0\leq s\lim_{k\to \infty }d\left(x_{n_{k+1}},fx^{*}\right)\leq 0$$

$$s\lim_{k\to \infty }d\left(x_{n_{k+1}},fx^{*}\right)=0$$

$$\lim_{k\to \infty }d\left(x_{n_{k+1}},fx^{*}\right)=0$$

$$\lim_{k\to \infty }x_{n_{k+1}}=fx^{*}$$

By uniqueness of limit and Complete b-metric space we have;

$$\lim_{k\to \infty }x_{n_{k+1}}=\lim_{k\to \infty }x_{n_{k}}=x^{*}=fx^{*}$$

$$x^{*}=fx^{*}$$

Hence, the fixed point of f is $x^{*}.$

**Remark:** *Theorem 3.1 provides a direct route to establishing the existence of a fixed point through initial conditions, Theorem 3.2 builds on this by examining the implications of sequential convergence and the relationships between subsequent points in the sequence, thus offering a more nuanced perspective on the fixed point's existence within the framework of contractive mappings in b-metric spaces.*

Corollary 3.1. Let f:X→X and α:X×X→[0,∞ ) are the predetermined mappings, while (X,d) is a complete metric space;

Let’s the given below principles are apply:-

(i)
f is α - ψ - ϕ Contractive mapping;
(ii)
f is triangular α -admissible mapping;
(iii) let $x_{0}\in X\;$ implies $\alpha (x_{0},fx_{0}$ ) ≥1 ; and
(iv) if $\left\{x_{n}\right\}$ in *X* implies $(x_{n},x_{n+1}$ ) ≥1 for every n∈ℕ and $x_{n}\to x^{*}\in X$ as n→∞,

For some subsequence $\left\{x_{n_{k}}\right\}$ of $\left\{x_{n}\right\}$ so that $\alpha (x_{n_{k}},x^{*}$ ) ≥1 for every k∈ℕ .

Then, a fixed point of f appears

Proof: If s is one, From Theorems **3.1** and **3.2** One finds this consequence.

Example 3.1. If *X* = {0,3,4}, d:X×X →[0,∞) is given by $d\left(x,y\right)={\left|x-y\right|}^{2},\left(X,d\right)$ is a complete b-metric space with s = 2, and also α:X×X →[0,∞) and f:X→X are given as follows; $\left\{ \begin{array}{ll} \alpha \left(x,y\right)=1, & \text{if}\;x,y\in \left\{\;0,3,4\right\}\;\\ \;\alpha \left(x,y\right)=0, & \text{other wise}\; \end{array}\right.$

f0=0,f3=3,f4=4

again, If ψ(t)=t and $\phi \left(t\right)=\frac{t}{3}$ , whenever ψ,ϕ:[0,∞)→[0,∞)∀t∈[0,∞) , then f is α−ψ−ϕ Contractive Mapping.

First, show that f is *α*-admissible mapping.

If x = 0, y=3 , α(0,3)≥1⇒α(f0,f3)=α(0,3)=1≥1

⇒f is *α*-admissible mapping.

Secondly, If x = 0, y=3andz=4 , the given equation holds.

⇒ f is triangular *α*-admissible.

Lastly, check that f is α−ψ−ϕ Contractive Mapping;

ψ(sd(fx,fy))=ψ(2d(f0,f3))=ψ(2d(0,3))=ψ(18)=18 (*)

$$\begin{array}{l} \psi \left(M\left(0,3\right)\right)-\phi \left(M\left(0,3\right)\right)=\psi \left(\frac{324+9n}{m}\right)-\phi \left(\frac{324+9n}{m}\right)\\ \;=\frac{324+9n}{m}-\frac{324+9n}{3m},\;\text{choose}\;n=\frac{1}{2}\;\text{and}\;m=1.\\ \;=\frac{324+\frac{9}{2}}{1}-\frac{324+\frac{9}{2}}{3}\\ \;=\frac{648+9}{2}-\frac{648+9}{6}=\frac{657}{2}-\frac{657}{6}\\ \;=\frac{657\;\left(3\right)-657}{6}=\frac{1,971-657}{6}\\ \;=\frac{1,314}{6}=219 \end{array}$$ (**)

From, (*) and (**), ψ(2d(f0,f3))≤ψ(M(0,3))−ϕ(M(0,3))

18 < 219

⇒ f is α−ψ−ϕ Contractive Mapping.

**Remark:** *In examining the distinctions between α−ψ contractive mappings and α−ψ−ϕ contractive mappings, it becomes evident that the latter provides a more flexible and nuanced approach to addressing fixed-point problems in b-metric spaces. While α−ψ contractive mappings focus primarily on the contraction properties defined by the functions α and ψ, the inclusion of the additional function ϕ in α−ψ−ϕ contractive mappings introduce a layer of complexity that allows for broader applications. This extension facilitates the accommodation of various contraction behaviors and interactions between the mappings, enhancing the potential for convergence. Furthermore, the α−ψ−ϕ framework enables the exploration of new classes of mappings and their properties, thus opening new avenues for research and applications in fields such as fixed-point theory, iterative methods, and mathematical modeling. By leveraging these advantages, researchers can derive more robust results and insights into the behavior of mappings in more complex environments, making α−ψ−ϕ contractive mappings a significant advancement over their α−ψ counterparts*.

## Conclusion

In conclusion, this research has introduced the concept of α−ψ−ϕ contractive mapping within b-metric spaces, expanding upon contraction theory principles. Through rigorous analysis and illustrative examples, we've highlighted its significance and applicability. Future directions include exploring stability in iterative methods, investigating connections with other mathematical areas, and extending the analysis to broader classes of spaces and mappings. Overall, this exploration of α−ψ−ϕ contractive mappings opens new avenues for research with broad potential implications.

## Authors contribution

All the authors equally contributed towards this work *.*

## Data Availability

The data is provided on request to the authors.
