# Supplementary material for: Exploring α−ψ−ϕ contractive mapping: novel fixed point theorems in complete b-metric spaces
Source: F1000Res. 2024 Dec 16;13:566. Originally published 2024 Jun 3. [Version 2] doi: 10.12688/f1000research.150979.2 (PMC11759888; doi:10.12688/f1000research.150979.2)
Supplement: Peer review history

# Reviewer report: Reviewer response for version 2

**DOI:** 10.5256/f1000research.173442.r350084

**Published:** 2025-01-24

**Authors:** Ashutosh Mishra

**Affiliations:** 1 Amity University, Lucknow, India

The work is purely theoretical, no practical application is mentioned. If some real-world problems were detailed then it would be beneficial to the scientific society. Although, the paper adequately describes and proves, as well, the abstracted notion. In Definition 3.1, condition 3.1 has some printing error. As a whole, I approve the work.

Is the work clearly and accurately presented and does it cite the current literature?

Yes

If applicable, is the statistical analysis and its interpretation appropriate?

Not applicable

Are all the source data underlying the results available to ensure full reproducibility?

No source data required

Is the study design appropriate and is the work technically sound?

Yes

Are the conclusions drawn adequately supported by the results?

Yes

Are sufficient details of methods and analysis provided to allow replication by others?

Yes

Reviewer Expertise:

Jungck Type Contractions, Fixed point theory and analysis.

I confirm that I have read this submission and believe that I have an appropriate level of expertise to confirm that it is of an acceptable scientific standard.

# Reviewer report: Reviewer response for version 2

**DOI:** 10.5256/f1000research.173442.r349199

**Published:** 2024-12-18

**Authors:** Tanakit Thianwan

**Affiliations:** 1 University of Phayao, Mueang Phayao District, Phayao, Thailand

No further comments.

Is the work clearly and accurately presented and does it cite the current literature?

Yes

If applicable, is the statistical analysis and its interpretation appropriate?

Yes

Are all the source data underlying the results available to ensure full reproducibility?

Yes

Is the study design appropriate and is the work technically sound?

Yes

Are the conclusions drawn adequately supported by the results?

Yes

Are sufficient details of methods and analysis provided to allow replication by others?

Yes

Reviewer Expertise:

Fixed point theory

I confirm that I have read this submission and believe that I have an appropriate level of expertise to confirm that it is of an acceptable scientific standard.

# Reviewer report: Reviewer response for version 1

**DOI:** 10.5256/f1000research.165597.r320358

**Published:** 2024-10-09

**Authors:** Konsalraj Julietraja, Agilan P

**Affiliations:** 1 Presidency University, Bengaluru, India; 2 Mathematics, St. Joseph's College of Engineering, Chennai, Tamil Nadu, India

The article "Exploring α−ψ−ϕ Contractive Mapping: Novel Fixed Point Theorems in Complete b-Metric Spaces" delves into advanced fixed point theory, focusing on the development of novel contractive conditions involving α, ψ, and ϕ mappings in the framework of complete b-metric spaces. The authors aim to generalize and extend classical fixed-point theorems, such as the Banach Contraction Principle, by introducing new types of contractive conditions, thus broadening the scope of potential applications in mathematics and its interdisciplinary fields. The article requires the following major revisions in order to make it suitable for indexing:

1. The authors should detail the contribution of the article in the abstract.
2. The authors should improve the Introduction section with the necessary references.
3. The authors should provide a clear definition of contractive mapping and α−ψ−ϕ contractive mapping:
4. The authors should justify the role of ϕ.
5. Theorem 3.1 - The statement is not clear. The authors should provide an unambiguous statement.
6. The authors should include the comparison between Theorem 3.1 and 3.2
7. The authors should compare α−ψ Contractive Mapping and α−ψ−ϕ Contractive Mapping and explore the advantages of α−ψ−ϕ Contractive Mapping.
8. The authors should cite the article entitled “Modified α-ψ-contractive mappings with applications Fixed Point Theory and Applications” 2013, 2013:151.
9. The authors should justify the relevance of the references 1, 6, 9,17 in their article.

Is the work clearly and accurately presented and does it cite the current literature?

Partly

If applicable, is the statistical analysis and its interpretation appropriate?

Not applicable

Are all the source data underlying the results available to ensure full reproducibility?

No source data required

Is the study design appropriate and is the work technically sound?

Partly

Are the conclusions drawn adequately supported by the results?

Partly

Are sufficient details of methods and analysis provided to allow replication by others?

No

Reviewer Expertise:

Applied Mathematics, Fixed point theory, Chemical Graph Theory

We confirm that we have read this submission and believe that we have an appropriate level of expertise to confirm that it is of an acceptable scientific standard, however we have significant reservations, as outlined above.

# Response

**Published:** 2024-10-14

**Authors:** Nasir Ali

**Affiliations:** Mathematics, COMSATS University Islamabad, Lahore, Punjab, Pakistan

Dear Reviewer,

 Thank you for your thoughtful and constructive feedback on our article, "Exploring α−ψ−ϕ Contractive Mapping: Novel Fixed Point Theorems in Complete b-Metric Spaces." We appreciate your insights, which will help enhance the clarity and impact of our work. Below, we address each of your major revision points in detail:

1. **Contribution in the Abstract**: We have revised the abstract to explicitly outline the key contributions of our article. It now clearly states how our novel contractive conditions extend classical fixed-point theorems and emphasizes the broader implications for mathematics and interdisciplinary applications.
2. **Improvement of the Introduction**: The introduction has been enhanced with additional references that provide context and support for our research. We have incorporated relevant literature to establish a stronger foundation for our work.
3. **Definition of Contractive Mappings**: We have added a clear and precise definition of contractive mappings, including a detailed explanation of α−ψ−ϕ contractive mappings. This section has been formulated to ensure that readers can easily understand the distinctions and relevance of these mappings within our framework.
4. **Justification of the Role of ϕ**: A new subsection has been included to justify the role of the function ϕ in our contractive mappings. We explain how its inclusion adds complexity and depth to the mapping conditions, enabling more generalized applications and insights into fixed-point behaviors.
5. **Clarification of Theorem 3.1**: We have rephrased Theorem 3.1 to provide a clearer and more unambiguous statement. The revised formulation emphasizes the key principles involved and their implications for the existence of fixed points.
6. **Comparison Between Theorem 3.1 and Theorem 3.2**: A comparison between Theorems 3.1 and 3.2 has been added to the manuscript. This section discusses the similarities and differences in the conditions and implications of each theorem, highlighting their respective contributions to fixed-point theory.
7. **Comparison of α−ψ and α−ψ−ϕ Contractive Mappings**: We have included a discussion comparing α−ψ and α−ψ−ϕ contractive mappings. This analysis explores the advantages of the α−ψ−ϕ framework in terms of flexibility and potential for broader applications in various mathematical contexts.
8. **Citation of Relevant Articles**: We have cited the article “Modified α-ψ-contractive mappings with applications Fixed Point Theory and Applications” (2013:151) as recommended. We believe that this reference complements our discussion and strengthens our literature review.
9. **Relevance of References 1, 6, 9, and 17**: Justification of References:
  Felhi, A., Aydi, H., & Zhang, D. (2016): This reference is critical as it discusses fixed points for α-admissible contractive mappings, which directly relates to the theoretical foundation of our work. The methodologies and results presented in this paper provide a comparative backdrop for our exploration of α−ψ−ϕ contractive mappings, emphasizing the evolution of fixed-point theory through varying mapping conditions.
  Güngör, G. D., & Altun, I. (2024): This article presents new fixed-point theorems applicable to quasi-metric spaces, showcasing the versatility and adaptability of fixed-point theory in different mathematical frameworks. Our inclusion of this reference serves to highlight the ongoing advancements in fixed-point theorems and their relevance to the broader mathematical community, particularly in how they relate to our findings in complete b-metric spaces.
  Ji, D., Yu, Y., & Li, C. (2024): The authors of this paper address fixed-point and endpoint theorems in convex b-metric spaces, which aligns closely with our study's context. This reference is particularly significant as it extends the applicability of fixed-point theory to multivalued mappings, reinforcing the importance of diverse mapping approaches in achieving generalizable results. It also helps position our work within a contemporary research landscape that seeks to unify various contractive conditions.
  Huang, M., Wang, X., Wang, Z., & Xu, Z. (2023): This research explores locally bi-Hölder continuous maps and their embeddings between Besov spaces, contributing to our understanding of continuity and its implications for fixed-point theorems. The techniques discussed in this paper may offer potential methodologies for further investigating the properties of α−ψ−ϕ contractive mappings. Including this reference enriches our discussion on the continuity conditions necessary for the application of fixed-point theorems in various mathematical settings.

We hope that these revisions address your concerns and enhance the clarity and quality of our article. Thank you once again for your valuable feedback, and we look forward to your further comments.

 Sincerely,

# Reviewer report: Reviewer response for version 1

**DOI:** 10.5256/f1000research.165597.r320355

**Published:** 2024-09-27

**Authors:** Tanakit Thianwan

**Affiliations:** 1 University of Phayao, Mueang Phayao District, Phayao, Thailand

**REPORT FOR THE PAPER**

 I reviewed the document and identified the following mistakes:

- In the keywords section, "maping" should be "mapping."
- In the definitions, some equations lack proper spacing between variables and symbols, making them harder to read.
- Page 4 of 11, line-2, Inconsistent use of semicolons, such as in "Let’s the given below principles are apply;" which should be rephrased for clarity.
- Page 4 of 11, line-2, "Let’s the given below principles are apply" should be "Let the following principles apply."
- The language used in the introduction is formal and appropriate for an academic paper. However, there are minor grammar issues, such as in the sentence "Through a meticulous synthesis of internet sources, scholarly publications, and pertinent literature, we establish the theoretical framework for this novel concept," where "internet sources" may not be the best fit for a research paper. Replacing it with "previous studies" or "existing research" might be more suitable.
- Ensure that all symbols and notation are consistent across the entire document. For example, double-check that the use of ψ and ϕ functions is consistent in both definitions and examples.
- Furthermore, there are many typos. The authors should be revised carefully.

Content Accuracy: The introduction provides a solid background but would benefit from a clearer and more concise explanation of how α-ψ-ϕ contractive mappings expand upon traditional contraction theory. It would be helpful to explain why the generalization to b-metric spaces is significant for real-world applications.

 Suggestion: Include a brief mention of practical applications of these mappings in areas like data science or machine learning to ground the mathematical theory in broader fields.

 In addition, the following references should be added.

 Baewnoi K, et al., 2024 (Ref 1).

 Chairatsiripong C, et al., 2023 (Ref 2)

 Yambangwai, D., et al., 2024 (Ref 3)

 Paimsang P, et al., 2023 (Ref 4)

 By citing these works,  helps establish the background and significance of introducing an alternative method, as the properties and applications of nonexpansive mappings in Banach spaces have strong implications for contractive mappings in b-metric spaces.

 In essence, these citations establish a comprehensive framework for fixed point theory and iterative methods, which is crucial for understanding and situating the novel contributions made by exploring α−ψ−ϕ contractive mappings in complete b-metric spaces. They showcase related iterative techniques, their convergence behaviors, and their applications across different spaces, thereby providing both a theoretical foundation and practical motivations for the new theorems being developed.

Is the work clearly and accurately presented and does it cite the current literature?

Yes

If applicable, is the statistical analysis and its interpretation appropriate?

Yes

Are all the source data underlying the results available to ensure full reproducibility?

Yes

Is the study design appropriate and is the work technically sound?

Yes

Are the conclusions drawn adequately supported by the results?

Yes

Are sufficient details of methods and analysis provided to allow replication by others?

Yes

Reviewer Expertise:

Fixed point theory

I confirm that I have read this submission and believe that I have an appropriate level of expertise to confirm that it is of an acceptable scientific standard, however I have significant reservations, as outlined above.

## References

[1] : A novel algorithm with an inertial technique for fixed points of nonexpansive mappings and zeros of accretive operators in Banach spaces. *AIMS Mathematics* .2024;9(3) : 10.3934/math.2024313 6424-6444 10.3934/math.2024313

[2] : Convergence theorem and convergence rate of a new faster iteration method for continuous functions on an arbitrary interval. *Miskolc Mathematical Notes* .2023;24(2) : 10.18514/MMN.2023.3978 10.18514/MMN.2023.3978

[3] : A parallel inertial SP-iteration monotone hybrid algorithm for a finite family of G-nonexpansive mappings and its application in linear system, differential, and signal recovery problems. *https://carpathian.cunbm.utcluj.ro/wp-content/uploads/carpathian_2024_40_2_535_557.pdf* .2024; 10.37193/CJM.2024.02.19 10.37193/CJM.2024.02.19

[4] : Mixed-type SP-iteration for asymptotically nonexpansive mappings in hyperbolic spaces. *Demonstratio Mathematica* .2023;56(1) : 10.1515/dema-2023-0113 10.1515/dema-2023-0113

# Response

**Published:** 2024-10-14

**Authors:** Nasir Ali

**Affiliations:** Mathematics, COMSATS University Islamabad, Lahore, Punjab, Pakistan

Dear Reviewer,

 Thank you for your thorough review and valuable feedback on our manuscript. We appreciate your constructive comments and have carefully addressed each one in our revised submission. Below is a summary of the revisions made in response to your suggestions:

1. **Keyword Correction**: We corrected the typo in the keywords section by changing "maping" to "mapping."
2. **Spacing in Equations**: We reviewed all equations and ensured that proper spacing between variables and symbols is applied for better readability.
3. **Clarity and Grammar**:
  - The sentence "Let’s the given below principles are apply" has been rephrased to "Let the following principles apply" for clarity.
  - We reviewed the manuscript for consistent use of semicolons and corrected them where needed to ensure uniformity and clarity in punctuation.
4. **Introduction Refinement**: As per your suggestion, we replaced "internet sources" with "previous studies" to maintain the formal tone appropriate for an academic paper. Additionally, we provided a clearer and more concise explanation of how α−ψ−ϕ contractive mappings expand upon traditional contraction theory, and why their generalization to b-metric spaces is significant for real-world applications.
5. **Consistency in Symbols and Notation**: We carefully reviewed the use of all symbols and notations throughout the document, including the ψ\psiψ and ϕ\phiϕ functions, and ensured consistency in both definitions and examples.
6. **Grammar and Typos**: We have carefully proofread the manuscript to eliminate any remaining typos and improve overall grammar.
7. **Content Accuracy and Practical Applications**: We revised the introduction to more clearly explain the importance of generalizing contraction theory to b-metric spaces and included a brief discussion on the practical applications of these mappings in fields such as data science and machine learning, grounding the mathematical theory in broader, real-world contexts.
8. **Addition of References**: We have added the suggested references:
  - Baewnoi K, et al., 2024 (Ref 1).
  - Chairatsiripong C, et al., 2023 (Ref 2).
  - Yambangwai, D., et al., 2024 (Ref 3).
  - Paimsang P, et al., 2023 (Ref 4).
  These references strengthen the theoretical framework and provide important background for understanding the significance of introducing α−ψ−ϕ\alpha-\psi-\phiα−ψ−ϕ contractive mappings in complete b-metric spaces. They also establish connections to related iterative techniques and their convergence behaviors.

We have uploaded the revised version of the manuscript, incorporating all these changes. We believe these revisions have improved the clarity, consistency, and overall quality of the paper, and we hope that it now meets the expectations of the review process.

 Thank you again for your insightful comments and suggestions.

 Sincerely,
